# Ways to improve biocides for metalworking fluid

**DOI:** 10.3934/microbiol.2021002

**Published:** 2021-01-18

**Authors:** Patrick Di Martino

**Affiliations:** Laboratoire ERRMECe, Cergy-Paris Université, 1 rue Descartes 95000 Neuville-sur-Oise, France

**Keywords:** Biocide, enhancer, booster, microorganism, metal working fluid

## Abstract

Metalworking fluids (MWF) are mainly emulsions of oil in water containing additives such as corrosion inhibitors, emulsifiers, defoamers, and biocides. Microbial contamination of MWF is almost systematic, and some of their constituents serve as nutrients for contaminating microorganisms. Biocides for MWF are protection products used to counter microbial contaminations and growth. Ideally, a biocide for MWF should have the following non-exhaustive criteria: have a broad-spectrum activity, be usable at low concentrations, be compatible with the formulation and the physical-chemical properties of MWF, be stable over time, retain its effectiveness in the presence of soiling, have no corrosive action on metals, present no danger to humans and the environment, be inexpensive. The future lies in the development of new molecules with biocidal activity corresponding to these ideal specifications, but in the meantime, it is possible to improve the performance of existing molecules currently on the market. Different strategies for potentiation of the activity of existing biocides are possible. The compatibility of the potentiation strategies with their use in metal working fluids is discussed.

## 1. Introduction

Biocidal products are substances or preparations for domestic or industrial use intended to destroy, repel or render harmless harmful organisms (fungi, bacteria, viruses, rodents, insects, etc.), to prevent their action or to combat them, by a chemical or biological action [1]. Biocidal products are subject to European regulation EU N° 528/2012 which harmonizes their placing on the market and their use within the European Union.

In the EU Regulation N ° 528/2012, 22 types of biocidal products (PT) divided into 4 groups are defined according to their use:

1. Group 1 (PT 1 to 5): disinfectants (human or animal hygiene, disinfection of surfaces, disinfection of drinking water, etc.).
2. Group 2 (PT 6 to 13): protection products (protection products for wood, construction materials, fluids for metalworking, etc.). This product group only corresponds to products intended to prevent microbial and algal growth.
3. Group 3 (PT 14 to 20): pest control products (rodenticides, insecticides, repellents, etc.).
4. Group 4 (PT 21 to 22): other biocidal products (anti-fouling products, fluids used for embalming and taxidermy).

For a biocide to enter the European market, it must be ensured that it is effective against the claimed target organisms, it does not induce unacceptable deleterious effects on non-target organisms and in particular on humans, but also that it does not induce resistance or cross resistance on target organisms. The undesirable effects of certain biocides which have been widely used for a long time, such as the isothiazolinone derivatives, have led to reduce their use and to better warning users of the risks associated with their exposure [2]. The intensive use of biocides can also lead to reductions in their effectiveness or even to reductions in the effectiveness of other chemicals [3]. The structure, activity of biocides as well as tolerance and bacterial resistance to biocides have already been reviewed [4]–[9]. In some bacteria such as methicillin-resistant *Staphylococcus aureus* (MRSA), a decrease in sensitivity to chlorhexidine gluconate is associated with a decrease in sensitivity to various antibiotics due to the activity of efflux pumps [10]. Impaired cell permeability to antimicrobials is a major mechanism of bacterial resistance to biocides and antibiotics [11]. Target modification or inactivation of biocides occur rarely and are limited to particular biocides such as triclosan or organomercurials, respectively [12],[13]. Phenotypic and physiological adaptation to particular growth patterns such as availability of certain nutrient substrates, reduced growth rate, nutrient limitation, or biofilm formation can alter the sensitivity of bacteria to biocides [9].

After a few reminders on biocides, a focus will be on the biocides used in the protection of metalworking fluids and the possibilities for improving their efficiency.

## 2. Biocides

### 2.1. Mechanism of action

In general, the action of a biocide against microorganisms takes place in three phases: the physical-chemical absorption of the biocide on the microbial surface, the penetration of the biocide into the cell and the action of the biocide on target sites [6],[7]. However, the penetration of the biocide into the microorganism is not always necessary for its action, which reduces the number of phases of action of some biocides. A biocide acts on one or more cellular targets such as the cytoplasmic membrane, or intracellular biological macromolecules (proteins, nucleic acids, etc.). As there is a diversity of the envelope structures of microorganisms (Gram-positive bacteria, Gram-negative bacteria, bacterial spores, Mycobacteria, yeasts, moulds, viruses) and a diversity of molecular structures of biocides, each biocide presents its own inactivation mechanism and spectrum of activity. The main mechanisms of microorganism inactivation by organic biocides and bacterial targets for biocides are presented in Table 1.

**Table 1.** Mechanisms of microorganism inactivation by different families of organic molecules with biocidal activity.

Types of Biocides	Mechanisms of action
Acids	Interaction with cell membranes
Active halogen compounds	Binding to -SH groups, inhibition and inactivation of proteins
Alcohols	Protein denaturation, dissolution of cell membranes
Aldehyde and compounds releasing formaldehyde	Binding to NH_2_ groups, inhibition and inactivation of proteins and nucleic acids
Biguanides	Interaction with cell membranes, inhibition and inactivation of proteins and nucleic acids
Cationic surfactants	Modification of membrane potential, destabilization of cell membranes
Isothiazolinones	Inhibition of Enzymes
Oxidizing or chlorine releasing compounds	Oxidation of cellular components, interaction with cell membranes, inactivation of proteins and nucleic acids
Phenolic compounds	Protein denaturation, alteration of cell membranes

### 2.2. Antibacterial activity tests of biocides

The bactericidal and bacteriostatic activities of biocides are conventionally determined against planktonic bacteria by determining a Minimum Inhibitory Concentration (MIC) and a Minimum Bactericidal Concentration (MBC). The MIC is the lowest concentration of the product that inhibits in 18 hours the visible multiplication of a bacterial suspension calibrated at 10^6^ Colony Forming Units (CFU)/mL [14]. The MBC is the lowest concentration of the product that reduces the viability of a calibrated initial bacterial population by a pre-determined reduction varying from 3 to 5 logs (3 to 5 decimal reductions) depending on the context [15]–[17]. The antibacterial activity of biocides can also be determined against bacteria attached to a surface and against bacteria inside biofilms. The ‘germ carrier’ method makes it possible to determine the activity of water-miscible liquid biocides towards bacteria artificially fixed on non-porous supports by drying [18]. To date, there is no standard technique for determining the activity of biocides solutions against biofilms. However, protocols are described in the literature, and guidance from public agencies are available [19]–[22].

In addition to the quantification of the antibacterial activity of a fluid containing a biocide, it is possible to have qualitative information on this activity using fluorescent markers of bacterial cell integrity (SYBR® Green II and propidium iodide) and markers of bacterial metabolic activity (ChemChrome V6 or 5-cyano-2,3-ditolyl tetrazolium chloride staining) [23],[24]. SYBR® Green is a fluorescent nucleic acid marker capable of entering all cells, regardless of their physiological state. Propidium iodide (PI) is a fluorescent nucleic acid dye that can penetrate only cells that have lost their membrane integrity (dead or structurally damaged cells). Upon excitation at 488 nm after SYBR® Green-PI double labelling, there can be a fluorescence resonance energy transfer from the SYBR® Green to the PI when these two fluorochromes are attached nearby one another on DNA. Under these conditions, the green fluorescence energy of SYBR® Green is absorbed by the PI, which can then fluoresce red. Cells whose membranes are compromised, and therefore permeable to PI, fluoresce in red, while cells with intact membranes, and therefore impermeable to PI, fluoresce in green. ChemChrome V6 reveals esterase activity in active bacterial cells. This non-fluorescent lipophilic molecule passively penetrates and accumulates in cells. In the intracellular compartment of active cells, it undergoes enzymatic hydrolysis by esterases, which forms the green fluorescent carboxyfluorescein product. Reduction of 5-cyano-2,3-ditolyl tetrazolium chloride (CTC) by the dehydrogenase activity of the respiratory chain induces the formation of the insoluble compound formazan that fluoresces red. The use of a combination of these fluorescent dies makes it possible to determine whether cells are inactivated which may be a reversible state and / or structurally altered which is generally an irreversible state which indicates cell death [25].

### 2.3. Biocides for metalworking fluids

Metal Working Fluids (MWF) serve several purposes: lubricating the interface between a tool and a metal surface, cooling, and removing debris from surfaces [26]. MWFs must have good stability over time. In addition to preparing an oil and water emulsion, the formulation of an MWF includes many additives such as amines, esters, corrosion inhibitors, emulsifiers, defoamers, and biocides. MWFs are used by diluting concentrated stock solutions with water. Since the concentrated solution represents only a few percent of the final product, the quality of the dilution water is essential to obtain a stable MWF. This water naturally brings contaminating microorganisms, as do all surfaces coming into contact with the MWF. These contaminating microorganisms can develop in the MWF in the form of suspended biomass but also on surfaces in contact with the MWF (reservoir wall, pipe interior and machine surface) in the form of fixed biomass and as floating biofilm at the surface of the fluid in reservoirs [26]–[28]. A microorganism inside a biofilm has a reduced sensitivity to biocides compared to the same microorganism in planktonic form [29],[30]. Among the additives used in the formulation of an MWF, the choice of biocides used, their concentration, their activity in the end-product are essential elements for the stability of MWF.

Ideally, a biocide for metalworking fluid should have the following non-exhaustive criteria: have a broad-spectrum activity, be usable at low concentrations, be compatible with the formulation and the physical-chemical properties of metalworking fluids, be stable over time, retain its effectiveness in the presence of soiling, have no corrosive action on metals, present no danger to humans and the environment, be inexpensive.

## 3. Microbial contamination of metalworking fluids

The selection and evaluation of the efficacy of the biocides used to ensure the stability of MWFs and limit bacterial growth is a critical point in their formulation [31]. Microbial contamination of water-miscible metalworking fluids is extremely common with culturable micro-organisms concentrations as high as 10^9^ CFU mL^−1^ [32]. The contaminating microbial populations evolve quantitatively and qualitatively during the lifetime of an MWF. This means that a biocidal system for MWF has to face constant but evolving challenges with diverse microorganisms for a long period of time. The constituents of MWF serve as nutrients for microbial growth. Some compounds of MWF are more sensitive to rapid microbial degradation than others, which induces non-uniform biodegradation of MWF [32],[33]. High rates of microbial degradation have been observed for fatty acids as well as for fatty acid amides. Fatty alcohol ethoxylates can also be degraded by bacteria isolated from a contaminated mineral oil based MWF emulsion. The nutrients in the fluid change as microorganisms consume carbon sources and form new growth substrates that can be used by other microorganisms by degrading ingredients in the MWF formulation. Thus, nitrite and nitrate are formed in MWF during the microbial degradation of monoethanolamine (MEA) [33]. Nitrates can then be reduced by different bacterial species like *Comamonas testosteroni* and *Pseudomonas putida*. In addition, new sources of organic and mineral nutrients are released after cell death and lysis over time.

The main agents of MWF deterioration are Gram-negative bacteria [31]–[35]. Pseudomonadaceae are strict aerobic organisms present consistently in emulsions with relatively moderate levels of contamination (up to 10^6^ CFU/mL) [31],[33]. When the biocontamination increases with a bacterial concentration of at least 10^8^ CFU/mL, facultative anaerobic organisms such as Enterobacteriaceae can become dominant with the presence of sulphur-producing bacteria at lower levels (10^3^–10^5^ CFU/mL). During the last stages of biodegradation, characterized by a drop in pH and an onset of phase shift of the emulsion, bacterial biodiversity increases and various Gram-negative bacteria can be isolated, such as *Acinetobacter*, *Achromobacter* and *Alcaligenes*. Many species of Gram-positive bacteria (*Micrococcus*, *Staphylococcus*, *Streptococcus* and *Bacillus*) and atypical Mycobacteria also called non-tuberculous are other common contaminants of MWF [32]–[37]. These fast-growing Mycobacteria are important members of MWF related biofilms. Yeasts and filamentous fungi are often present in contaminated MWF but at low concentrations (10^2^–10^4^ CFU/mL). *Fusarium oxysporum* has been identified in MWF emulsions based on mineral oils [33]. In addition to inducing biodeterioration of MWFs, contaminating microorganisms present a health risk to operators working in contact with them [38],[39].

The main objective of the use of biocides is to limit microbial growth within MWFs, but certain contaminants, such as Pseudomonadaceae, are capable of degrading these biocides [34],[40],[41], in particular when they develop in the form of biofilm [42].

## 4. How to potentiate the activity of a biocide? Application to biocides for metalworking fluids

The first approach to potentiate the activity of a biocide is to combine several biocides with synergistic activity [43],[44]. It also helps overcome the biocide resistance of some microorganisms. Combinations of biocides have been described as exhibiting a synergistic effect, i.e. the efficacy of the combined antimicrobials is greater than the sum of the individual compounds [45].

The choice of biocides that can be used for metalworking is limited to the regulatory list of products for the protection of working or cutting fluids (TP13, products to fight against microbial alterations in fluids used for working or cutting metal, glass or other materials) (Table 2).

While the biocides on this list provide some level of protection to MWFs, some of them have significant limitations. Thus, triazine-based biocides are not very effective against bacteria reducing sulphates (Sulphate Reducing Bacteria, SRB) and against Mycobacteria [46]. In addition, these biocides act by releasing formaldehyde which is very volatile. Biocides based on BIT (1,2-benzisothiazol-3(2H)-one) have the advantage of having a mechanism of action independent of formaldehyde. On the other hand, they are not very effective against Pseudomonadaceae and Mycobacteria and are inactivated in the event of contamination by SRBs. Oxazolidines are effective against Pseudomonadaceae, SRBs, and Mycobacteria. An increase in the action spectrum of oxazolidines is obtained by association with OIT (2-octyl-2H-isothiazol-3-one) with antifungal properties. MBM (N, N′-methylenebismorpholine) exhibits a broad spectrum of antifungal and antibacterial activity including against Mycobacteria. However, MBM can induce eye irritation and skin sensitization.

**Table 2.** List of TP13 products authorized in the European Union to fight against microbial alterations in fluids used for working or cutting metal, glass or other materials. Abbreviations of biocides are given in brackets.

Substance name	EC number	CAS number
alpha, alpha′, alpha″-trimethyl-1,3,5-triazine-1,3,5(2H,4H,6H)-triethanol (HPT)	246-764-0	25254-50-6
1,2-benzisothiazol-3(2H)-one (BIT)	220-120-9	2634-33-5
(benzyloxy)methanol	238-588-8	14548-60-8
Biphenyl-2-ol	201-993-5	90-43-7
1,3-bis(hydroxymethyl)-5,5-dimethylimidazolidine-2,4-dione (DMDMH)	229-222-8	6440-58-0
2-butyl-benzo[d]isothiazol-3-one (BBIT)	420-590-7	876403
cis-1-(3-chloroallyl)-3,5,7-triaza-1-azoniaadamantane chloride (cis CTAC)	426-020-3	51229-78-8
Chlorocresol	200-431-6	59-50-7
2,2-dibromo-2-cyanoacetamide (DBNPA)	233-539-7	10222-01-2
4,4-dimethyloxazolidine	257-048-2	51200-87-4
7a-ethyldihydro-1H,3H,5H-oxazolo[3,4-c]oxazole (EDHO)	231-810-4	7747-35-5
(ethylenedioxy)dimethanol (Reaction products of ethylene glycol with paraformaldehyde (EGForm))	222-720-6	3586-55-8
Glutaral (Glutaraldehyde)	203-856-5	111-30-8
2,2′,2″-(hexahydro-1,3,5-triazine-1,3,5-triyl)triethanol (HHT)	225-208-0	1029713
3-iodo-2-propynylbutylcarbamate (IPBC)	259-627-5	55406-53-6
N-methyl-1,2-benzisothiazolin-3-one (MBIT)	Not available	2527-66-4
Methenamine 3-chloroallylochoride (CTAC)	223-805-0	4080-31-3
2-methyl-2H-isothiazol-3-one (MIT)	220-239-6	2682-20-4
3,3′-methylenebis[5-methyloxazolidine] (Oxazolidin/MBO)	266-235-8	66204-44-2
N-(3-aminopropyl)-N-dodecylpropane-1,3-diamine (Diamine)	219-145-8	2372-82-9
N,N′-methylenebismorpholine (MBM)	227-062-3	5625-90-1
Mixture of 5-chloro-2-methyl-2H-isothiazol-3-one and 2-methyl-2H-isothiazol-3-one (Mixture of CMIT/MIT)		55965-84-9
2-octyl-2H-isothiazol-3-one (OIT)	247-761-7	26530-20-1
2-Phenoxyethanol	204-589-7	122-99-6
Pyridine-2-thiol-1-oxide-, sodium salt (Sodium pyrithione)	223-296-5	3811-73-2
Silver chloride	232-033-3	7783-90-6
Sodium 2-biphenylate	205-055-6	132-27-4
Tetrahydro-1,3,4,6-tetrakis(hydroxymethyl)imidazole[4,5-d]imidazole-2,5′1H,3H)-dione (TMAD)	226-408-0	5395-50-6

Thus, it appears advantageous to include in the formulation of MWF, one or more constituents having an action of potentiation of the activity of the biocides present. These constituents must share several characteristics of the specifications for MWF biocides and in particular, be usable at low concentrations, be compatible with the formulation and physicochemical properties of MWF, be stable over time, not have a corrosive action on metals, do not present a danger to humans and the environment, be inexpensive.

Different avenues for enhancers/boosters of biocide activity, and/or alternatives to biocides exist in the literature. Examples of enhancer/booster agents are presented in Table 3.

**Table 3.** Different categories of enhancers/boosters of biocide activity and alternatives to biocides.

Category of enhancer/booster and alternative agents	Examples
Organic permeabilizing agents	Ethylenediamine tetra-acetic acid (EDTA)
	Ethylene diamine disuccinate (EDDS)
	Polyethyleneimine (PEI)
	meso-2,3-dimercatosuccinic acid (DMSA)
Metals	Lithium
	Copper
Organic and inorganic acids	Acetic acid
	Citric acid
	Phosphoric acid
	Sorbic acid
Multifunctional agents for self-protection	Glycerol
	Levulinic acid

### 4.1. Organic permeabilizing agents

The first type of molecule that can potentiate the action of biocides corresponds to organic permeabilizing agents acting on microbial membranes. Among these molecules are ethylenediamine tetra-acetic acid (EDTA), polyethyleneimine (PEI), and meso-2,3-dimercatosuccinic acid (DMSA). In the study published by Alakomi et al., EDTA, PEI, and DMSA were tested alone or in the presence of a biocide (benzalkonium chloride) or different antibiotics for an effect on the growth of *Pseudomonas* and *Stenotrophomonas*, two Gram-negative bacteria [47]. Each of the three compounds increases membrane permeability, the addition of MgCl_2_ to the reaction medium decreases or abolishes this effect depending on the permeabilizer and its concentration. The addition of DMSA induces acidification of the medium, an effect not encountered with the other two permeabilizers. PEI increases the sensitivity of *Pseudomonas* to different hydrophobic antibiotics, this effect is not found or partially found in other bacterial species. PEI increases the effectiveness of benzalkonium chloride against *Pseudomonas* in the planktonic state (in suspension), but EDTA does not. EDTA and DMSA alone have an inhibiting effect on biofilm formation of different bacterial species tested, but not PEI. EDTA also enhances the bacteriostatic activity of glutaraldehyde and tetrakis(hydroxymethyl)phosphonium sulfate (THPS) against planktonic SRB [48],[49]. Ethylene diamine disuccinate (EDDS) biodegradable chelator improves glutaraldehyde efficacy against SRB biofilms both as preventive or curative treatment [50],[51].

There is a family of synthetic polyethyleneimines (PEIs) which are weakly basic polycationic aliphatic polymers. PEI-based hydrogels have been approved by the FDA as surgical dressings which underscores their safety. Linear PEIs (LPEIs) or branched PEIs (RPEIs) target the cytoplasmic membrane of bacteria and yeast, causing rapid microbicidal properties [52]. LPEIs distinguish between membranes of mammals and those of bacterial models, while RPEIs lack selectivity and can induce toxicity in humans.

In the study published by Lefebvre et al., The action of EDTA alone and in the presence of biocides was tested against biofilms formed by *P. aeruginosa* and by the Gram-positive species *Staphylococcus aureus* [20]. EDTA alone at a concentration of 20 mM induces a significant decrease in the cultivable bacterial concentration in biofilms of *P. aeruginosa* (factor 100) and *S. aureus* (factor 20). The addition of the same concentration of EDTA to different formulations of commercial biocides for medical use (Prontosan, Octeniline, Providone iodine) conducted to a reduction in the cultivable bacterial concentration in biofilms of the two species varying from 95 to more than 99%. In addition, during these tests, biocidal concentrations lower than the recommended usage concentrations were used in order to demonstrate the synergistic action between EDTA and the biocides.

This type of molecules with permeabilizing activity is therefore of potential interest as an enhancer/booster of the activity of biocides in metal treatment fluids, even if it seems necessary to test several of them with respect to different species of bacteria contaminating these fluids. Their use could increase the antibacterial activity of MWF formulations and could decrease the concentration of biocides in these formulations.

### 4.2. Metals

The second type of enhancer/booster of biocidal activity is also a permeabilizer but of a different nature [53]. It is lithium. In the study published by Di Maiuta et al., the enhancer/booster activity of lithium provided in various ways in solutions of biocides (formulations containing isothiazolinones associated with formaldehyde liberators or isothiazolinones associated with glutaraldehyde) was tested against strains of *Pseudomonas putida* and *Methylobacterium extorquens* resistant to formaldehyde. Lithium was supplied in a solution of calcium carbonate either via neutralization-dispersion or in the form of lithium carbonate. Regardless of its mode of supply, lithium had an activity to enhance/boost the activity of the biocidal solutions tested. What is particularly interesting in this study was that the enhancer/booster activity was observed with respect to bacterial strains which have developed resistance to certain biocides and that in the formulations used, biocides also used in the fluids for the treatment of metals were present (CMIT/MIT). Lithium would act by disrupting the Na^+^/H^+^ antiport system which would lead to a toxic accumulation of Na^+^ ions intracellularly and to a disturbance of the electrochemical gradient of protons at the level of the bacterial membrane. The membrane would then be depolarized and lose its integrity. While there is a literature on lithium toxicity, it appears that the risks associated with its use generally appear to be limited [54].

Other metals such as copper exhibit in themselves an antimicrobial activity but above all are capable of potentiating the action of biocides used in MWFs by a synergistic action [55],[56]. The use of Cu^2+^ with biocides reduces the effective concentration of biocide and thus minimizes the toxicity of its use. However, the toxicity of copper and its implication in various human pathologies make its use in industrial environment uncertain [57].

### 4.3. Organic and inorganic acids

Adding organic acids to the formulation of MWFs could also provide benefits in terms of increased antimicrobial activity. Organic acids affect microbial activity by cytoplasmic acidification, and by accumulation of the dissociated acid anion to toxic levels intracellularly [58]. The acidic pH in the internal cell damages or alters the functionality of enzymes, structural proteins and DNA [59]. Organic acids are only active against microorganisms in their undissociated form, the only form capable of crossing the cell membrane. Once in the cell, organic acids dissociate and can act. In addition, hydrophobic organic acids such as sorbic acid increase membrane permeability and cause interference with membrane proteins [60]. Organic acids with shorter carbon chains like acetic acid and sorbic acid have better antimicrobial activity than acids with long carbon chains [61].

Organic acids are widely used in the food industry as food preservatives and as decontamination agents for poultry carcasses or meat products. They are recognized as safe substances and approved as food preservatives by the European Union, WHO and FDA [62]. Organic acids are considered to be naturally occurring preservatives. Acetic acid exhibits antibacterial activity against different strains of *Salmonella* [59]. On the other hand, cells in the stationary phase are 100 times more resistant to the action of acetic acid than cells in the exponential phase of growth. In addition, the sensitivity of *Salmonella* to acetic acid is strain dependent. Interestingly, acetic acid has a synergistic action with various natural phenols with antimicrobial properties such as Thymol or Carvacrol [63]. However, the conditions of use of acetic acid in the food industry are very different from the conditions of use of MWF. Citric acid has antibacterial activity by chelating metals, with increased activity compared to monocarboxylic acids such as lactic acid [64]. Phosphoric acid is an inorganic acid that exhibits more effective antibacterial activity against Gram-positive cocci of the species *Enterococcus faecalis* than citric acid [65]. Concentrations and effective contact times are lower for phosphoric acid than for citric acid. Sorbic acid is an unsaturated fatty acid widely used for its antibacterial and antifungal properties as a preservative in foodstuffs, cosmetics and pharmaceuticals. Sorbate inhibits a large panel of Gram-positive and Gram-negative bacteria, both aerobic and anaerobic [66]. Interestingly, sorbate inhibits the germination of bacterial spores but in a strain dependent manner. The antimicrobial activity of sorbic acid depends on many environmental and physicochemical factors such as the activity of water, pH, temperature, the composition of the product containing it (presence of salts, antioxidants, sugars, antimicrobials). The concentrations of sorbic acid required to inhibit bacterial growth vary widely depending on the genus or species of bacteria. Thus, it is 10 mg/L for *Lactobacillus* but 10,000 mg/L for *Clostridium*. Potassium sorbate is only able to delay the growth of certain moulds (*Aspergillus ochraceus* and *Penicillium aurantiogriseum*) [67], and certain resistant strains develop in the presence of high concentrations of potassium sorbate (> 12000 ppm) [68]. Thus, one of the problems associated with the use of organic acids as food preservatives is the gradual decrease in sensitivity or even the acquisition of resistance for certain microorganisms [69]. Furthermore, sorbic acid can exhibit genotoxicity *in vitro* on different cell lines and *in vivo* in some animal models [70]. However, no carcinogenic activity has been reported for long-term use of sorbic acid and its potassium salt in mice and rats. Sorbic acid is not genotoxic in itself, but products of its oxidation are toxic. In contrast, potassium sorbate is irritating to the skin, eyes and respiratory tract, but does not exhibit any proven toxicity at usual concentrations [71].

An important limitation to the use of organic acids to potentiate the action of biocides in MWFs is that they can constitute substrates of choice for microbial metabolism and are therefore very easily biodegraded [72],[73]. So, some organic acids like citric acid enter the tricarboxylic acid cycle (Krebs cycle), where they are converted into CO_2_ and H_2_O. Citrate is an essential substrate for microbial respiration [73]. Benzoic acid can be converted to protocatechuate before being transformed into CO_2_ and H_2_O by the same route [72]. Lactic acid is first oxidized to pyruvate which is oxidized to acetyl-CoA and then transformed into citrate which is then degraded to CO_2_ and H_2_O.

### 4.4. Multifunctional agents for self-protection

In the cosmetics industry, there has been a development of so-called self-protected products in recent years [74]. Thus, traditional preservatives are replaced by other cosmetic ingredients which are used mainly as functional constituents of a cosmetic product, but which significantly contribute to the total preservative function, since they have antimicrobial properties. Levulinic acid (4-oxo-pentanoic acid) is an example of this type of multifunctional ingredient. Addition to emulsions of levulinic acid at relatively low concentrations (≤ 0.3%) in addition to other antimicrobial agents provides broad spectrum antimicrobial properties against *Pseudomonas aeruginosa*, *Escherichia coli*, *Staphylococcus aureus*, *Candida albicans* and *Aspergillus niger* [74]. However, in a recent study, levulinic acid alone was found to be ineffective in protecting a cosmetic formulation [68]. Levulinic acid can be combined with the ionic surfactant SDS (sodium dodecyl sulphate) to potentiate its action against different types of microorganisms [75]. In this case, the most effective concentrations are in the range of 2 to 3%. The combination of levulinic acid and SDS promotes the detachment of bacterial cells from the biofilm and kills detached cells by the action of the undissociated form of levulinic acid. Synergistic action between SDS and lactic acid for their antibacterial activity has also been demonstrated [76],[77]. Levulinic acid and SDS are recognized by the FDA as safe for use in food.

Quite recently, a new generation of MWF composed of glycerol and water rather than mineral oil and water has been described [78]. Glycerol has lubricating, corrosion protection and viscosity modulating properties [78]. Glycerol, like all short chain alcohols, exhibits a bacteriostatic and fungistatic effect if its concentration is high enough. Thus, these glycerol and water based MWFs do not contain conventional biocides. A glycerol concentration of at least 30 to 35% is necessary to limit microbial growth in an acceptable manner [79],[80]. Nevertheless, glycerol present at lower concentration can serve as a nutrient and improve microbial development.

## 5. Conclusions

Metalworking fluids are multifunctional, their stability in use is an essential property to take into account in their formulation. To ensure the stability of MWFs against microbial contamination, the use of biocides comes up against a choice limited by the regulations, and imperfect antimicrobial properties of the authorized molecules. Several strategies for optimizing the antimicrobial properties of MWFs are possible: seeking a synergistic action between several biocides; developing self-protected MWFs; adding enhancer/booster of biocidal activity to the formulation of MWF. For this, it is necessary to conduct an in-depth study of the types of ingredients used in the formulation of MWFs, taking into account potential antimicrobial properties for the choice of some of them (acids, bases, surfactants, etc.), and consider adding new ingredients compatible with the regulations and the final formulation of MWFs. When evaluating new ingredients with antimicrobial properties or enhancer/booster of antimicrobial activity, this activity must be determined against planktonic cells but also against the same cells inside biofilms. A gain in antimicrobial activity against planktonic and biofilm microorganisms provides a double advantage in terms of stability of an MWF under conditions of use.
